# Role of ferroptosis in atrial fibrillation: a review

**DOI:** 10.3389/fphar.2025.1362060

**Published:** 2025-02-06

**Authors:** Shaowei Fan, Yuanhui Hu, Jingjing Shi

**Affiliations:** ^1^ Lugouqiao Second Community Health Service Center, China Aerospace Science & Industry Corporation 731 Hospital, Beijing, China; ^2^ Department of Cardiological Medicine, China Academy of Chinese Medical Sciences Guang’anmen Hospital, Beijing, China

**Keywords:** ferroptosis, atrial fibrillation, lipid peroxidation, myocardial infarction, autophagy

## Abstract

Cardiovascular disease remains the leading cause of mortality, with atrial fibrillation emerging as one of the most common conditions encountered in clinical practice. However, its underlying mechanisms remain poorly understood, prompting ongoing research. Ferroptosis, a recently discovered form of regulated cell death characterized by lipid peroxidation and disrupted cellular redox balance leading to cell death due to iron overload, has attracted significant attention. Since its identification, ferroptosis has been extensively studied in various contexts, including cancer, stroke, myocardial ischemia/reperfusion injury, and heart failure. Growing evidence suggests that ferroptosis may also play a critical role in the onset and progression of atrial fibrillation, though research in this area is still limited. This article provides a concise overview of the potential mechanisms by which ferroptosis may contribute to the pathogenesis of atrial fibrillation.

## 1 Overview of ferroptosis

Iron is essential for numerous vital biological processes, including DNA synthesis, transfer, and the production of enzymes critical for cellular cycles and biogenesis. As a result, iron metabolism is a key biochemical process, tightly regulated by genetic mechanisms. Within cells, iron primarily exists in heme, ferritin, and iron-sulfur clusters in mitochondrial proteins. Iron regulatory proteins 1 and 2 (IRP1 and IRP2) act as intracellular sensors, regulating iron transport and storage. Transferrin (TFR) is the main protein responsible for iron uptake from the extracellular environment. Additionally, ferritin plays a crucial role in sequestering free iron, thereby protecting cells from ferroptosis.

Ferroptosis is a distinct form of cell death, different from apoptosis, characterized by iron-dependent lipid peroxidation and non-apoptotic oxidative damage. It involves both enzymatic mechanisms (mediated by lipoxygenases, LOXs) and non-enzymatic mechanisms (via the Fenton reaction), leading to iron-driven lipid peroxidation. The enzymatic pathway is primarily regulated by glutathione peroxidase 4 (GPX4), which reduces lipid hydroperoxides to lipid alcohols, thereby preventing excessive lipid peroxidation and ferroptosis. When phospholipid membranes rich in polyunsaturated fatty acids (PUFAs) undergo excessive peroxidation due to Fe^2+^ activation, and antioxidant defenses are overwhelmed, iron accumulates, triggering ferroptosis (Hassannia et al., 2019; Conrad and Pratt, 2019). The presence of PUFAs in membranes enhances fluidity, supporting cellular adaptation to external changes. A decrease in GPX4 levels promotes ferroptosis. Studies have shown that reduced GPX4 expression and subsequent iron buildup contribute to ferroptosis, a process implicated in various cardiovascular conditions, including myocardial infarction, ischemia/reperfusion injury, heart failure, arrhythmias, and other heart diseases (Hong et al., 2022).

Several key steps in lipid oxidation contribute to the initiation of ferroptosis. Initially, acyl-CoA synthetase long-chain family member 4 (ACSL4) catalyzes the attachment of coenzyme A to long-chain polyunsaturated fatty acids (PUFAs) within the phospholipid membrane, activating them for subsequent reactions. Additionally, PUFAs may undergo esterification by lysophosphatidylcholine acyltransferase 3 (LPCAT3), further modifying the lipid composition of the membrane.

Both ACSL4 and LPCAT3 are key regulators of lipid metabolism and play a role in iron activation. Intracellular iron activation can trigger membrane lipid peroxidation, which, in turn, initiates ferroptosis. This peroxidation process leads to several detrimental effects, including disrupted protein function, altered membrane fluidity, increased membrane permeability, structural damage, membrane rupture, cytotoxicity, and ultimately, ferroptotic cell death. These events highlight the intricate relationship between lipid metabolism, iron homeostasis, and the induction of ferroptosis (Tang et al., 2021).

During ferroptosis, lipid peroxidation generates various byproducts, including lipid hydroperoxides (LOOHs), malondialdehyde (MDA), 4-hydroxynonenal (4-HNE), and other reactive aldehydes (Tang et al., 2021). Mitochondria are a major source of reactive oxygen species (ROS), which play a crucial role in driving ferroptosis. Elevated ROS levels can lead to DNA damage, protein denaturation, and lipid peroxidation.

ROS include singlet oxygen and three types of free radicals: hydroxyl radical (OH^−^), superoxide anion (O^2−^), and hydrogen peroxide (HO_2_·). Among these, OH^−^ is the most chemically reactive, generated through the Fenton reaction between Fe^2+^ and hydrogen peroxide. This highly reactive hydroxyl radical directly initiates non-enzymatic lipid peroxidation, a process catalyzed by iron.

Iron ions can enhance the activity of enzymes involved in lipid peroxidation and oxygen homeostasis, such as LOX and EGLN proline hydroxylases. These enzymes contribute to lipid peroxidation and the generation of reactive species. To counteract iron-induced cellular damage, lipid antioxidants like α-tocopherol or endostatin-1, along with iron chelators, can effectively mitigate these harmful effects (Kotschi et al., 2022; Zilka et al., 2017).

It is important to note that our understanding of ferroptosis and its complex molecular mechanisms is still evolving, with ongoing research shedding light on the intricate processes driving this form of cell death.

Iron accumulation in the body can increase ROS production and trigger inflammation (Masaldan et al., 2019). Free iron, which forms the labile iron pool (LIP), is characterized by weak chelation, redox activity, and its availability for the Fenton reaction (Jamnongkan et al., 2017). LIP instability is primarily caused by increased iron absorption, reduced storage capacity, ferritin degradation, or transferrin dysfunction, all of which disrupt redox balance and promote ferroptosis in myocardial cells (Lou et al., 2021).

Ferroptosis involves various organelles, including mitochondria, lysosomes, and the endoplasmic reticulum. This form of cell death can lead to the loss of specific leukocyte subsets, impairing their immune functions, and may also affect non-leukocytic cells. Different types of cell death release distinct damage-associated molecular patterns (DAMPs), triggering varied immune-related inflammatory responses. As a result, ferroptosis is considered an immunogenic process (Minagawa et al., 2020).

Morphologically, ferroptotic cells exhibit compromised membrane integrity, cytoplasmic swelling, preserved nuclear size, altered electron density, and significant mitochondrial damage. This includes rupture of the outer mitochondrial membrane, increased membrane density, reduced mitochondrial volume, decreased cristae in the inner membrane, and the absence of NADH. Notably, ATP levels remain stable during ferroptosis, as this process relies on ATP production, not caspase activation, for initiation (Xu et al., 2019). Ferroptosis can also propagate to adjacent cells in waves through an osmotic mechanism (Riegman et al., 2020; Katikaneni et al., 2020).

## 2 Overview of atrial fibrillation

Atrial fibrillation (AF) is a common arrhythmia with a wide range of contributing factors, including age, male gender, obesity, genetics, lifestyle choices, metabolic syndrome, hypertension, coronary artery disease, valvular heart disease, heart failure, diabetes, hyperthyroidism, chronic kidney disease, and chronic obstructive pulmonary disease (Schnabel et al., 2009; Wasmer et al., 2017; Tomaszuk-Kazberuk et al., 2020). These conditions often induce structural changes in the atria, such as atrial enlargement, increased fibrosis, elevated epicardial adipose tissue, autonomic nervous system dysfunction, systemic inflammation, impaired myocardial contractile function, and alterations in myocardial cell structure (Vlachos et al., 2016).

AF affects over 30 million people globally (Chugh et al., 2014), representing approximately 2%–3.4% of the population (Zoni-Berisso et al., 2014; Kjerpeseth et al., 2021; Williams et al., 2020). In Europe, the prevalence is about 2.1%, with an annual incidence of 1.3 per 1,000 individuals. The economic burden of AF is considerable, impacting individuals, families, and societies, with annual treatment costs ranging from 450 to 3,000 euros per patient (Williams et al., 2020).

In the United States, over five million individuals have AF, with a prevalence of around 8% in those aged 65 and older (Colilla et al., 2013; Piccini et al., 2012). Annual treatment costs range from $2,000 to $14,200 per patient, contributing to a total expenditure exceeding $28 billion annually (Dieleman et al., 2020). AF significantly increases the risk of ischemic stroke, pulmonary embolism, heart failure, and mortality (Alonso et al., 2021). Patients with AF face a 50%–90% higher risk of mortality compared to those without the condition, with female patients potentially having a worse prognosis than males (Benjamin et al., 1998).

AF is primarily classified by the duration of episodes. Paroxysmal AF refers to episodes that resolve within 7 days, while persistent and permanent AF require medical intervention to manage or terminate the arrhythmia (Nattel, 2013). Treatment options for AF include oral anticoagulants for stroke prevention and surgical interventions. Significant progress has been made in the management of AF, driven by advances in understanding its mechanisms, pathophysiology, and surgical techniques. However, ongoing research is focused on determining whether these treatments offer long-term survival benefits for AF patients.

AF is characterized by key features such as atrial enlargement, myocardial cell hypertrophy, and increased extracellular matrix content in the atrial myocardium. These changes facilitate the formation of an electrical conduction loop that sustains the arrhythmia (Pandit and Jalife, 2013). Atrial premature beats often originate from the myocardial sleeve of the pulmonary vein, triggering reentry and leading to AF (Khan, 2004). In younger patients without structural heart abnormalities, rapid, localized activity inducing pulmonary vein arrhythmia may be the primary trigger for paroxysmal AF (Voigt et al., 2014). In contrast, older patients are more likely to develop persistent or permanent AF due to the combined effects of atrial tissue remodeling and metabolic disorders (Lau et al., 2016; Platonov et al., 2011).

Adverse cardiac remodeling, changes in ion channel expression and function, electrical remodeling, autonomic nervous system dysfunction, and disturbances in calcium homeostasis are well-established contributors to the development and progression of AF (Brunner et al., 2017; Guichard et al., 2020). Structural changes can directly or indirectly lead to atrial electrical abnormalities, triggering ectopic events that culminate in AF. However, determining whether these structural changes are a cause or consequence of AF remains challenging.

Key pathophysiological mechanisms include myocardial lipid peroxidation, fibrosis, inflammation, oxidative stress, and ferroptosis, all of which contribute to AF progression (Protchenko et al., 2021; Tang et al., 2019). However, the precise underlying pathogenesis is still under investigation, and no consensus has yet been reached. Atrial fibrosis is an essential feature of atrial structural remodeling, driven by factors that stimulate fibroblast proliferation and differentiation into myofibroblasts, resulting in excessive extracellular matrix (ECM) production. The predominant components of this ECM are type I and type III collagen, which contribute to the development of AF (Li et al., 2021). This process leads to ultrastructural changes in the heart, including widened vascular gaps and increased ECM deposition.

Atrial fibrosis can be categorized into two main types: reparative and reactive fibrosis. Reparative fibrosis occurs as a secondary response to myocardial cell loss, such as after a myocardial infarction. In contrast, reactive fibrosis is primarily associated with cardiac inflammation and excessive pressure load. Based on its location, reactive fibrosis can be further classified into interstitial or perivascular fibrosis (Kong et al., 2014).

Fibrosis is essential for maintaining cardiac structural integrity but disrupts normal electrical conduction. Fibroblasts and myofibroblasts form gap junctions with myocardial cells via Connexin proteins (Kohl and Gourdie, 2014; Ongstad and Kohl, 2016). However, their membrane potential is lower than that of atrial myocardial cells, which reduces action potential conduction velocity and maximum depolarization, slowing transverse but accelerating longitudinal conduction (Nguyen et al., 2014). This pathological coupling enhances automatic depolarization and promotes reentry circuits, contributing to AF (Nattel, 2018).

This review will explore the potential role of ferroptosis in the pathogenesis of AF.

## 3 Possible mechanisms of atrial fibrillation caused by ferroptosis

**FIGURE 1:**
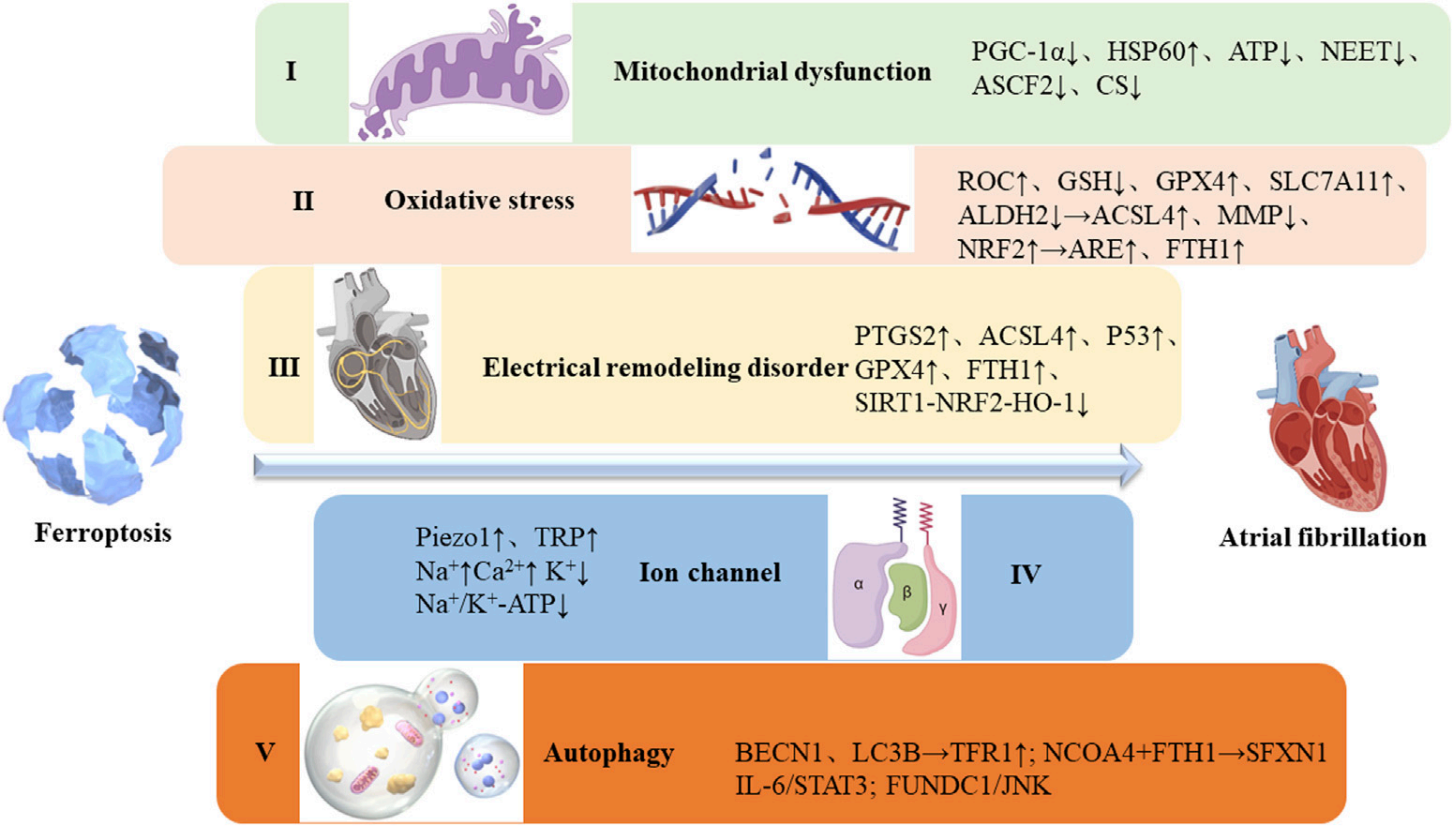
Possible mechanisms of atrial fibrillation caused by ferroptosis. I. Ferroptosis leads to mitochondrial dysfunction, disrupting ion gradients and membrane potential, ultimately altering cardiac electrical conduction and promoting arrhythmias; II. Oxidative stress results from an imbalance between the antioxidant and oxidative systems, significantly impacting iron homeostasis; III. Electrical remodeling is characterized by SR calcium overload, elevated cytosolic Ca^2+^ levels, reduced expression of slow inward calcium channels, increased rectifier potassium current, and altered expression of junction proteins; IV. Ion channels are regulated by ferroptosis, influencing their functional activity; V. Autophagy plays a key role in ferritin degradation and upregulation of TfR1 expression, increasing intracellular iron levels and promoting ferroptosis.

Activation of the renin-angiotensin-aldosterone system (RAAS), inflammation, oxidative stress, apoptosis, and autonomic imbalance are interconnected factors that contribute to the persistence of AF (Nattel et al., 2020; Van Wagoner and Chung, 2018). Ferroptosis, a form of regulated cell death, has been observed in various cell types, particularly in myocardial tissue under pathological conditions (Zheng and Conrad, 2020). This article explores the role of ferroptosis in AF pathogenesis, focusing on mitochondrial dysfunction, oxidative stress, electrical remodeling disturbances, ion channel dysfunction, autophagy, and other related factors (Figure 1).

### 3.1 Ferroptosis and mitochondrial dysfunction

The onset of AF is closely linked to myocardial dysfunction, driven by electrophysiological abnormalities and disturbances in ion exchange, which lead to electrical remodeling and arrhythmias caused by ectopic pacing. Emerging research suggests a potential connection between these electrical disturbances and mitochondrial dysfunction (Muszyński and Bonda, 2021). Mitochondria, essential for ATP production through the electron transport chain, are particularly vulnerable to ferroptosis, an organelle-specific form of cell death.

Cell membrane proteins regulate ion flux across the membrane, orchestrating vital biochemical processes. During ferroptosis, mitochondrial dysfunction disrupts ion gradients and membrane potential, leading to altered cardiac electrical conduction and the development of arrhythmias (Galaris et al., 2019). However, the precise mechanisms behind these processes require further investigation.

Mitochondria make up approximately 30% of the volume of myocardial cells and are essential for energy production, supplying about 90% of the energy required for cardiac contraction (Schaper et al., 1985; Harris and Das, 1991). Mitochondrial dysfunction can significantly impair both systolic and diastolic heart function. Studies have shown that such dysfunction disrupts ATP production, leading to the accumulation of ROS, which in turn disturbs calcium homeostasis and membrane excitability in myocardial cells, contributing to the development of AF (Clark and Mach, 2017).

Peroxisome proliferator-activated receptor γ coactivator-1α (PGC-1α) is a crucial nuclear transcription coactivator involved in mitochondrial biogenesis, energy metabolism, and the regulation of oxidative stress and inflammation (Chandrasekaran et al., 2015). Inhibition of PGC-1α results in mitochondrial dysfunction, oxidative stress, and intracellular calcium overload, ultimately triggering AF (Sahin et al., 2011; Summermatter et al., 2013). Patients with atrial fibrillation often show mitochondrial alterations, including abnormal ATP levels, elevated HSP60 expression, mitochondrial ATP depletion, reduced respiration and membrane potential, and abnormal morphology. These changes mirror those seen in other cardiac conditions such as myocardial infarction, heart failure, dilated cardiomyopathy, ischemic heart disease, hypertensive heart disease, and diabetic cardiomyopathy (Guzmán et al., 2014; Eirin et al., 2014). Ferroptosis is marked by mitochondrial constriction, loss of cristae, and outer membrane disruption (Kagan et al., 2020). Irregular mitochondrial morphology involves fragmentation (Chen et al., 2010), swelling, decreased density, cristae disarray (Cogliati et al., 2013), and disintegration (Guzmán et al., 2014). These structural changes impair mitochondrial respiratory function, hinder ATP synthesis, and compromise cardiac contractility. Additionally, prolonged mitochondrial calcium buffering delays calcium uptake, further disrupting mitochondrial respiration (Jeganathan et al., 2017; Wiersma et al., 2019).

Ferroptosis is induced through the mitochondrial voltage-dependent anion channel, activation of mitogen-activated protein kinase, and inhibition of the cystine/glutamate transporter (Xie et al., 2016). Mitochondria are central to iron synthesis, utilization, and degradation, thus maintaining iron homeostasis and supporting both material and energy metabolism. They regulate iron sensitivity across various metabolic pathways, with proteins such as ferritin, mitochondrial ferritin 1/2, and NEET playing key roles in iron regulation. Additionally, ASCF2 and CS are involved in mitochondrial lipid metabolism, while glutamine and other metabolic pathways are also influenced by mitochondrial function. Therefore, understanding mitochondrial responses during ferroptosis is crucial for clarifying its role in the initiation and progression of AF.

### 3.2 Ferroptosis and oxidative stress

Oxidative stress results from an imbalance between antioxidant and oxidative systems, significantly affecting iron homeostasis. The Fenton reaction is the primary source of oxidative stress in ferroptosis (Shen et al., 2018; Wang et al., 2018).

A key feature of oxidative stress is the elevation of ROS, which occurs when the body’s antioxidant defenses are insufficient to neutralize oxidizing substances. This leads to the accumulation of free radicals and other oxidative species, causing cellular damage. Oxidative stress is a well-established factor in the onset and progression of numerous diseases. Research shows that iron overload promotes glutathione (GSH) depletion, ROS production, lipid peroxidation, and the synthesis of ferroptosis markers such as GPX4 and the cystine-glutamate antiporter (SLC7A11). It also inhibits ACSL4 and reduces mitochondrial membrane potential (MMP). Ferroptosis can exacerbate mitochondrial oxidative stress through the NRF2-ARE pathway (Chen et al., 2022). Furthermore, iron overload induces lipid peroxidation, mitochondrial ROS production, and further decreases MMP.

MiR-23a-3p targets and regulates SLC7A11, promoting oxidative stress and ferroptosis, which contribute to the progression of AF (Liu et al., 2022). The key to mitigating oxidative stress injury lies in upregulating antioxidant-related genes and proteins, such as FTH1, GPX4, SLC7A11, and various antioxidant enzymes, to restore redox homeostasis (Stockwell et al., 2020).

GPX4 is a critical antioxidant enzyme that inhibits lipid peroxidation and reduces ROS accumulation (Thomas et al., 1990). When ROS levels rise or cystine levels decrease, cellular GSH levels drop, impairing GPX4 activity and promoting ferroptosis (Li et al., 2020a; Seiler et al., 2008). Studies have identified a correlation between GPX4 variants, especially rs713041, and cardiovascular diseases (Strauss et al., 2018; Admoni et al., 2019). Additionally, GPX4 variants may be linked to the onset of postoperative AF, along with changes in myocardial GPX4 content and activity (Berdaweel et al., 2022). These findings above indicate that ferroptosis could play a role in the initiation and progression of AF, potentially serving as a prognostic marker.

Mitochondrial aldehyde dehydrogenase (ALDH2) inhibits ACSL4, a key enzyme in lipid peroxidation, thereby preventing ferroptosis and improving cardiac systolic function (Zhu et al., 2022a). Conversely, alcohol is a known risk factor for AF with the ALDH2 heterozygous defect allele (*1/*2), who regularly consume alcohol, are at increased risk of AF due to slower alcohol metabolism and enhanced ferroptosis (Yamashita et al., 2022).

### 3.3 Ferroptosis and electrical remodeling disorder

The formation of arrhythmogenic lesions may be driven by diastolic calcium leakage from the sarcoplasmic reticulum (SR), caused by hyperphosphorylation of the ryanodine receptor (RyR). This promotes delayed depolarization and triggers activity that favors AF (Chan et al., 2019). Both reentry and rapid focal activity contribute to frequent atrial depolarization and electrical remodeling, characterized by reduced conduction velocity and a shortened atrial refractory period, which sustain AF (Lenaerts et al., 2009). Electrical remodeling is linked to SR calcium overload, elevated cytoplasmic Ca^2+^ levels, reduced expression of inward calcium channels, increased rectifier potassium current, and altered connexin expression (Wang et al., 2017).

Excessive ethanol consumption significantly elevates ferroptosis-promoting proteins (e.g., PTGS2, ACSL4, P53) and reduces anti-ferroptosis proteins (e.g., GPX4, FTH1) in the atrium, leading to substantial atrial damage. This damage includes increased susceptibility to AF, impaired atrial conduction, atrial enlargement, and heightened fibrosis markers (Yu et al., 2023). The SIRT1-Nrf2-HO-1 signaling pathway may provide therapeutic insights by inhibiting ferroptosis and protecting the atrium from injury.

### 3.4 Ferroptosis and ion channel

Recent research on ferroptosis has highlighted its regulatory effects on ion channels, which play a critical role in the development of AF. Cardiac rhythm generation is closely linked to the functional dynamics of ion channels.

During ferroptosis, lipid peroxides accumulate on the cell membrane, increasing surface tension and activating Piezo1 and TRP channels (Hirata et al., 2023). This lipid oxidation also enhances the permeability of cations such as Na^+^ and Ca^2+^, leading to their efflux from the cell. As a result, intracellular Na^+^ and Ca^2+^ concentrations rise, while K^+^ levels decrease. Additionally, membrane lipid oxidation reduces the activity of Na^+^/K^+^-ATPase, further disrupting monovalent cation gradients. This study emphasizes the crucial role of altered cation permeability—mediated by Piezo1, TRP channels, and Na^+^/K^+^-ATPase activity—in the initiation of ferroptosis.

During episodes of atrial fibrillation, progressive atrial enlargement is a key feature closely associated with atrial mechanical overload. In human atrial fibroblasts, at least two stretch-activated ion channels (SACs) are involved: Piezo1, a non-selective cation channel activated directly by mechanical stretch, and BKCa, a calcium-dependent, potassium-selective channel (Emig et al., 2021). In individuals with non-persistent atrial fibrillation, both the activity and expression of Piezo1 are significantly increased, suggesting its predominant role over other channels compared to sinus rhythm. In response to atrial mechanical stimulation, BKCa activity rises as a secondary response to Piezo1. In contrast, patients with persistent atrial fibrillation exhibit a reduction in BKCa activity. Additionally, the upregulation of TRP channels is implicated in both electrical and structural cardiac remodeling. TRP channels, which are non-selective cation channels with varying calcium permeability, consist of six subtypes expressed in different cell types (Yue et al., 2015). TRP channels contribute to endothelial cell apoptosis and cardiac fibrosis through fibroblast differentiation, playing a role in the onset and progression of various cardiovascular diseases (Yue et al., 2011). Multiple TRP subtypes have been shown to participate in atrial electrical remodeling in AF patients, though through different mechanisms (Yue et al., 2015). A study examining leukocyte TRP channels found significantly higher expression in patients with nonvalvular AF compared to controls (Düzen et al., 2017), suggesting that Piezo1 and TRP channels may serve as critical targets for ferroptosis, promoting the onset and progression of AF.

A study investigating sinoatrial node function in mice with chronic iron overload found that inhibition of the Ca_(V1.3)_ channel initially reduced the density of L-type calcium currents (I_(Ca,L)_), causing a rightward shift in the voltage activation curve of I_(Ca,L)_ (Rose et al., 2011). These changes led to a decrease in spontaneous action potential generation in sinoatrial node cells, resulting in bradycardia, prolonged PR intervals, cardiac blocks, and AF.

In conclusion, ferroptosis-induced modulation of ion channels may contribute to the development of AF.

### 3.5 Ferroptosis and autophagy

The ferroptosis inducer elastin can trigger autophagy through ROS generation. Autophagy plays a key role in regulating ferritin degradation and TFR1 expression, leading to increased intracellular iron levels and promoting ferroptosis (Park and Chung, 2019). This process involves a range of autophagic proteins that facilitate the degradation of damaged or excess cellular components. Double-membrane vesicles encapsulate these components, which then fuse with lysosomes for degradation (Klionsky, 2008; Yorimitsu and Klionsky, 2005). Autophagy serves both as an essential biological process, regulated by specific genes, and as a stress-responsive survival mechanism that may also contribute to cell death (Liu et al., 2013). Key autophagic proteins include BECN1 and light chain 3 β (LC3B). By engulfing ferritin through phagocytosis, autophagy elevates intracellular free iron levels, further promoting ferroptosis (Manz et al., 2016). Notably, the expression of BECN1 in valvular AF may offer a promising therapeutic target for future treatments of AF (Liu et al., 2021).

In a study of patients with postoperative atrial fibrillation (Garcia et al., 2012), significant accumulation of autophagic vesicles and lipofuscin deposits was observed in atrial tissue. Additionally, LC3B levels were reduced, indicating selective damage during the autophagic process. These findings suggest the presence of ultrastructural atrial remodeling associated with autophagy-related damage.

Nuclear receptor coactivator 4 (NCOA4) interacts directly with FTH1, facilitating the transport of ferritin to the autophagosome for degradation. This process triggers phagocytosis and ferritin breakdown, releasing large amounts of free iron. The resulting increase in cytoplasmic Fe^2+^ enhances the expression of exoflavin (SFXN1) on the mitochondrial membrane, promoting the transfer of Fe^2+^ into mitochondria (Dowdle et al., 2014; Bellelli et al., 2016). This leads to mitochondrial ROS production and ferroptosis, contributing to cardiac damage (Li et al., 2020b).

The IL-6/STAT3 signaling pathway regulates NCOA4-mediated ferritin phagocytosis, promoting ferroptosis in cardiomyocytes (Zhu et al., 2023). In a rapid atrial pacing (RAP)-induced AF animal model, activation of the IL-6/STAT3 pathway was observed in both peripheral blood and liver (Yaegashi et al., 2016). This activation increases fibrinogen expression, triggers the extrinsic prothrombin activation pathway, and induces a hypercoagulable state in AF.

Additionally, under Paraquat exposure, Protein 1 containing the Fun14 domain (FUNDC1)/JNK-mediated ferroptosis may contribute to cardiac and mitochondrial damage (Peng et al., 2022). Phosphorylation of FUNDC1 at the Ser17 site activates mitochondrial autophagy (Zhu et al., 2022b), a key process for eliminating dysfunctional or excess mitochondria and maintaining mitochondrial quality control (Pickles et al., 2018; Chang et al., 2021; Sun et al., 2022). Previous studies have highlighted the significant role of mitochondrial autophagy dysfunction in atrial muscle cells of patients with AF (Bravo-San Pedro et al., 2017). FUNDC1, a recently identified mitochondrial autophagy receptor, interacts directly with LC3B, a microtubule-associated protein (Chen et al., 2016; Lv et al., 2017).

Despite limited research on ferroptosis in AF, its pathogenesis, experimental validation, and drug mechanisms remain areas for extensive investigation. Therapeutic approaches targeting ferroptosis in atrial fibrillation may draw from research in other systemic diseases. Potential agents include experimental compounds like erastin and RSL3, drugs such as statins and artemisinin, ionizing radiation, and cytokines like IFNγ and TGF-β1, which may offer avenues for reversing AF (Chen et al., 2021).

## 4 Conclusion

Currently, surgical interventions such as radiofrequency ablation and cryoablation, along with pharmaceutical treatments like antiarrhythmic drugs and oral anticoagulants, remain the primary approaches for managing AF. However, significant gaps remain in our understanding of the underlying mechanisms and potential therapeutic targets for this condition. The recent recognition of ferroptosis has opened a promising new avenue for both the diagnosis and treatment of various diseases.

This article reviews the relationship between AF and ferroptosis, exploring how ferroptosis contributes to the condition through key mechanisms such as mitochondrial dysfunction, oxidative stress, electrical remodeling, ion channel abnormalities, and autophagy. By examining these pathways, the review aims to inform future research efforts and provide insights into the development of novel diagnostic and therapeutic strategies for atrial fibrillation.

## References

[B1] Admoni S. N. Santos-Bezerra D. P. Perez R. V. Patente T. A. Monteiro M. B. Cavaleiro A. M. (2019). Glutathione peroxidase 4 functional variant rs713041 modulates the risk for cardiovascular autonomic neuropathy in individuals with type 1 diabetes. Diab Vasc. Dis. Res. 16 (3), 297–299. 10.1177/1479164118820641 30599773

[B2] Alonso A. Almuwaqqat Z. Chamberlain A. (2021). Mortality in atrial fibrillation. Is it changing? Trends Cardiovasc Med. 31 (8), 469–473. 10.1016/j.tcm.2020.10.010 33127438 PMC8076338

[B3] Bellelli R. Federico G. Matte A. Colecchia D. Iolascon A. Chiariello M. (2016). NCOA4 deficiency impairs systemic iron homeostasis. Cell Rep. 14 (3), 411–421. 10.1016/j.celrep.2015.12.065 26776506

[B4] Benjamin E. J. Wolf P. A. D'Agostino R. B. Silbershatz H. Kannel W. B. Levy D. (1998). Impact of atrial fibrillation on the risk of death: the Framingham Heart Study. Circulation 98 (10), 946–952. 10.1161/01.cir.98.10.946 9737513

[B5] Berdaweel I. A. Hart A. A. Jatis A. J. Karlan N. Akhter S. A. Gaine M. E. (2022). A genotype-phenotype analysis of glutathione peroxidase 4 in human atrial myocardium and its association with postoperative atrial fibrillation. Antioxidants (Basel) 11 (4), 721. 10.3390/antiox11040721 35453406 PMC9026099

[B6] Bravo-San Pedro J. M. Kroemer G. Galluzzi L. (2017). Autophagy and mitophagy in cardiovascular disease. Circ. Res. 120 (11), 1812–1824. 10.1161/CIRCRESAHA.117.311082 28546358

[B7] Brunner S. Herbel R. Drobesch C. Peters A. Massberg S. Kääb S. (2017). Alcohol consumption, sinus tachycardia, and cardiac arrhythmias at the Munich octoberfest: results from the Munich beer related electrocardiogram workup study (MunichBREW). Eur. Heart J. 38 (27), 2100–2106. 10.1093/eurheartj/ehx156 28449090 PMC5837309

[B8] Chan Y. H. Chang G. J. Lai Y. J. Chen W. J. Chang S. H. Hung L. M. (2019). Atrial fibrillation and its arrhythmogenesis associated with insulin resistance. Cardiovasc Diabetol. 18 (1), 125. 10.1186/s12933-019-0928-8 31558158 PMC6761716

[B9] Chandrasekaran K. Anjaneyulu M. Inoue T. Choi J. Sagi A. R. Chen C. (2015). Mitochondrial transcription factor A regulation of mitochondrial degeneration in experimental diabetic neuropathy. Am. J. Physiol. Endocrinol. Metab. 309 (2), E132–E141. 10.1152/ajpendo.00620.2014 25944881 PMC4504935

[B10] Chang X. Lochner A. Wang H. H. Wang S. Zhu H. Ren J. (2021). Coronary microvascular injury in myocardial infarction: perception and knowledge for mitochondrial quality control. Theranostics 11 (14), 6766–6785. 10.7150/thno.60143 34093852 PMC8171103

[B11] Chen G. H. Song C. C. Pantopoulos K. Wei X. L. Zheng H. Luo Z. (2022). Mitochondrial oxidative stress mediated Fe-induced ferroptosis via the NRF2-ARE pathway. Free Radic. Biol. Med. 180, 95–107. 10.1016/j.freeradbiomed.2022.01.012 35045311

[B12] Chen H. Vermulst M. Wang Y. E. Chomyn A. Prolla T. A. McCaffery J. M. (2010). Mitochondrial fusion is required for mtDNA stability in skeletal muscle and tolerance of mtDNA mutations. Cell 141 (2), 280–289. 10.1016/j.cell.2010.02.026 20403324 PMC2876819

[B13] Chen M. Chen Z. Wang Y. Tan Z. Zhu C. Li Y. (2016). Mitophagy receptor FUNDC1 regulates mitochondrial dynamics and mitophagy. Autophagy 12 (4), 689–702. 10.1080/15548627.2016.1151580 27050458 PMC4836026

[B14] Chen X. Kang R. Kroemer G. Tang D. (2021). Broadening horizons: the role of ferroptosis in cancer. Nat. Rev. Clin. Oncol. 18 (5), 280–296. 10.1038/s41571-020-00462-0 33514910

[B15] Chugh S. S. Havmoeller R. Narayanan K. Singh D. Rienstra M. Benjamin E. J. (2014). Worldwide epidemiology of atrial fibrillation: a global burden of disease 2010 study. Circulation 129 (8), 837–847. 10.1161/CIRCULATIONAHA.113.005119 24345399 PMC4151302

[B16] Clark A. Mach N. (2017). The crosstalk between the gut microbiota and mitochondria during exercise. Front. Physiol. 8, 319. 10.3389/fphys.2017.00319 28579962 PMC5437217

[B17] Cogliati S. Frezza C. Soriano M. E. Varanita T. Quintana-Cabrera R. Corrado M. (2013). Mitochondrial cristae shape determines respiratory chain supercomplexes assembly and respiratory efficiency. Cell 155 (1), 160–171. 10.1016/j.cell.2013.08.032 24055366 PMC3790458

[B18] Colilla S. Crow A. Petkun W. Singer D. E. Simon T. Liu X. (2013). Estimates of current and future incidence and prevalence of atrial fibrillation in the U.S. adult population. Am. J. Cardiol. 112 (8), 1142–1147. 10.1016/j.amjcard.2013.05.063 23831166

[B19] Conrad M. Pratt D. A. (2019). The chemical basis of ferroptosis. Nat. Chem. Biol. 15 (12), 1137–1147. 10.1038/s41589-019-0408-1 31740834

[B20] Dieleman J. L. Cao J. Chapin A. Chen C. Li Z. Liu A. (2020). US health care spending by payer and health condition, 1996-2016. JAMA 323 (9), 863–884. 10.1001/jama.2020.0734 32125402 PMC7054840

[B21] Dowdle W. E. Nyfeler B. Nagel J. Elling R. A. Liu S. Triantafellow E. (2014). Selective VPS34 inhibitor blocks autophagy and uncovers a role for NCOA4 in ferritin degradation and iron homeostasis *in vivo* . Nat. Cell Biol. 16 (11), 1069–1079. 10.1038/ncb3053 25327288

[B22] Düzen I. V. Yavuz F. Vuruskan E. Saracoglu E. Poyraz F. Göksülük H. (2017). Leukocyte TRP channel gene expressions in patients with non-valvular atrial fibrillation. Sci. Rep. 7 (1), 9272. 10.1038/s41598-017-10039-0 28839241 PMC5571177

[B23] Eirin A. Lerman A. Lerman L. O. (2014). Mitochondrial injury and dysfunction in hypertension-induced cardiac damage. Eur. Heart J. 35 (46), 3258–3266. 10.1093/eurheartj/ehu436 25385092 PMC4258226

[B24] Emig R. Knodt W. Krussig M. J. Zgierski-Johnston C. M. Gorka O. Groß O. (2021). Piezo1 channels contribute to the regulation of human atrial fibroblast mechanical properties and matrix stiffness sensing. Cells 10 (3), 663. 10.3390/cells10030663 33809739 PMC8002259

[B25] Galaris D. Barbouti A. Pantopoulos K. (2019). Iron homeostasis and oxidative stress: an intimate relationship. Biochim. Biophys. Acta Mol. Cell Res. 1866 (12), 118535. 10.1016/j.bbamcr.2019.118535 31446062

[B26] Garcia L. Verdejo H. E. Kuzmicic J. Zalaquett R. Gonzalez S. Lavandero S. (2012). Impaired cardiac autophagy in patients developing postoperative atrial fibrillation. J. Thorac. Cardiovasc Surg. 143 (2), 451–459. 10.1016/j.jtcvs.2011.07.056 21885071

[B27] Guichard J. B. Naud P. Xiong F. Qi X. L'Heureux N. Hiram R. (2020). Comparison of atrial remodeling caused by sustained atrial flutter versus atrial fibrillation. J. Am. Coll. Cardiol. 76 (4), 374–388. 10.1016/j.jacc.2020.05.062 32703507

[B28] Guzmán M. G. Báez A. L. Lo Presti M. S. Domínguez R. Córdoba R. Bazán C. (2014). Functional and structural alterations of cardiac and skeletal muscle mitochondria in heart failure patients. Arch. Med. Res. 45 (3), 237–246. 10.1016/j.arcmed.2014.03.003 24657595

[B29] Harris D. A. Das A. M. (1991). Control of mitochondrial ATP synthesis in the heart. Biochem. J. 280 (Pt 3), 561–573. 10.1042/bj2800561 1837214 PMC1130493

[B30] Hassannia B. Vandenabeele P. Vanden Berghe T. (2019). Targeting ferroptosis to iron out cancer. Cancer Cell 35 (6), 830–849. 10.1016/j.ccell.2019.04.002 31105042

[B31] Hirata Y. Cai R. Volchuk A. Steinberg B. E. Saito Y. Matsuzawa A. (2023). Lipid peroxidation increases membrane tension, Piezo1 gating, and cation permeability to execute ferroptosis. Curr. Biol. 33 (7), 1282–1294.e5. 10.1016/j.cub.2023.02.060 36898371

[B32] Hong M. Rong J. Tao X. Xu Y. (2022). The emerging role of ferroptosis in cardiovascular diseases. Front. Pharmacol. 13, 822083. 10.3389/fphar.2022.822083 35153792 PMC8826236

[B33] Jamnongkan W. Thanan R. Techasen A. Namwat N. Loilome W. Intarawichian P. (2017). Upregulation of transferrin receptor-1 induces cholangiocarcinoma progression via induction of labile iron pool. Tumour Biol. 39 (7), 1010428317717655. 10.1177/1010428317717655 28671021

[B34] Jeganathan J. Saraf R. Mahmood F. Pal A. Bhasin M. K. Huang T. (2017). Mitochondrial dysfunction in atrial tissue of patients developing postoperative atrial fibrillation. Ann. Thorac. Surg. 104 (5), 1547–1555. 10.1016/j.athoracsur.2017.04.060 28760472

[B35] Kagan V. E. Tyurina Y. Y. Sun W. Y. Vlasova I. I. Dar H. Tyurin V. A. (2020). Redox phospholipidomics of enzymatically generated oxygenated phospholipids as specific signals of programmed cell death. Free Radic. Biol. Med. 147, 231–241. 10.1016/j.freeradbiomed.2019.12.028 31883467 PMC7037592

[B36] Katikaneni A. Jelcic M. Gerlach G. F. Ma Y. Overholtzer M. Niethammer P. (2020). Lipid peroxidation regulates long-range wound detection through 5-lipoxygenase in zebrafish. Nat. Cell Biol. 22 (9), 1049–1055. 10.1038/s41556-020-0564-2 32868902 PMC7898270

[B37] Khan R. (2004). Identifying and understanding the role of pulmonary vein activity in atrial fibrillation. Cardiovasc Res. 64 (3), 387–394. 10.1016/j.cardiores.2004.07.025 15537491

[B38] Kjerpeseth L. J. Igland J. Selmer R. Ellekjær H. Tveit A. Berge T. (2021). Prevalence and incidence rates of atrial fibrillation in Norway 2004-2014. Heart 107 (3), 201–207. 10.1136/heartjnl-2020-316624 32820014 PMC7815897

[B39] Klionsky D. J. (2008). Autophagy revisited: a conversation with Christian de Duve. Autophagy 4 (6), 740–743. 10.4161/auto.6398 18567941

[B40] Kohl P. Gourdie R. G. (2014). Fibroblast-myocyte electrotonic coupling: does it occur in native cardiac tissue? J. Mol. Cell Cardiol. 70 (100), 37–46. 10.1016/j.yjmcc.2013.12.024 24412581 PMC4001130

[B41] Kong P. Christia P. Frangogiannis N. G. (2014). The pathogenesis of cardiac fibrosis. Cell Mol. Life Sci. 71 (4), 549–574. 10.1007/s00018-013-1349-6 23649149 PMC3769482

[B42] Kotschi S. Jung A. Willemsen N. Ofoghi A. Proneth B. Conrad M. (2022). NFE2L1-mediated proteasome function protects from ferroptosis. Mol. Metab. 57, 101436. 10.1016/j.molmet.2022.101436 34999280 PMC8814388

[B43] Lau D. H. Schotten U. Mahajan R. Antic N. A. Hatem S. N. Pathak R. K. (2016). Novel mechanisms in the pathogenesis of atrial fibrillation: practical applications. Eur. Heart J. 37 (20), 1573–1581. 10.1093/eurheartj/ehv375 26578197

[B44] Lenaerts I. Bito V. Heinzel F. R. Driesen R. B. Holemans P. D'hooge J. (2009). Ultrastructural and functional remodeling of the coupling between Ca2+ influx and sarcoplasmic reticulum Ca2+ release in right atrial myocytes from experimental persistent atrial fibrillation. Circ. Res. 105 (9), 876–885. 10.1161/CIRCRESAHA.109.206276 19762679

[B45] Li C. Y. Zhang J. R. Hu W. N. Li S. N. (2021). Atrial fibrosis underlying atrial fibrillation (Review). Int. J. Mol. Med. 47 (3), 9. 10.3892/ijmm.2020.4842 33448312 PMC7834953

[B46] Li J. Cao F. Yin H. L. Huang Z. J. Lin Z. T. Mao N. (2020a). Ferroptosis: past, present and future. Cell Death Dis. 11 (2), 88. 10.1038/s41419-020-2298-2 32015325 PMC6997353

[B47] Li N. Wang W. Zhou H. Wu Q. Duan M. Liu C. (2020b). Ferritinophagy-mediated ferroptosis is involved in sepsis-induced cardiac injury. Free Radic. Biol. Med. 160, 303–318. 10.1016/j.freeradbiomed.2020.08.009 32846217

[B48] Liu A. Jia K. Liang H. Jin Q. (2021). Comprehensive analysis of autophagy-related genes and patterns of immune cell infiltration in valvular atrial fibrillation. BMC Cardiovasc Disord. 21 (1), 132. 10.1186/s12872-021-01939-1 33706714 PMC7948357

[B49] Liu D. Yang M. Yao Y. He S. Wang Y. Cao Z. (2022). Cardiac fibroblasts promote ferroptosis in atrial fibrillation by Secreting exo-miR-23a-3p targeting SLC7A11. Oxid. Med. Cell Longev. 29, 3961495. 10.1155/2022/3961495 PMC9168132 35677105

[B50] Liu Y. Shoji-Kawata S. Sumpter R. M. Jr Wei Y. Ginet V. Zhang L. (2013). Autosis is a Na+,K+-ATPase-regulated form of cell death triggered by autophagy-inducing peptides, starvation, and hypoxia-ischemia. Proc. Natl. Acad. Sci. U. S. A. 110 (51), 20364–20371. 10.1073/pnas.1319661110 24277826 PMC3870705

[B51] Lou J. S. Zhao L. P. Huang Z. H. Chen X. Y. Xu J. T. Tai W. C. S. (2021). Ginkgetin derived from Ginkgo biloba leaves enhances the therapeutic effect of cisplatin via ferroptosis-mediated disruption of the Nrf2/HO-1 axis in EGFR wild-type non-small-cell lung cancer. Phytomedicine 80, 153370. 10.1016/j.phymed.2020.153370 33113504

[B52] Lv M. Wang C. Li F. Peng J. Wen B. Gong Q. (2017). Structural insights into the recognition of phosphorylated FUNDC1 by LC3B in mitophagy. Protein Cell 8 (1), 25–38. 10.1007/s13238-016-0328-8 27757847 PMC5233613

[B53] Manz D. H. Blanchette N. L. Paul B. T. Torti F. M. Torti S. V. (2016). Iron and cancer: recent insights. Ann. N. Y. Acad. Sci. 1368 (1), 149–161. 10.1111/nyas.13008 26890363 PMC4870095

[B54] Masaldan S. Bush A. I. Devos D. Rolland A. S. Moreau C. (2019). Striking while the iron is hot: iron metabolism and ferroptosis in neurodegeneration. Free Radic. Biol. Med. 133, 221–233. 10.1016/j.freeradbiomed.2018.09.033 30266679

[B55] Minagawa S. Yoshida M. Araya J. Hara H. Imai H. Kuwano K. (2020). Regulated necrosis in pulmonary disease. A focus on necroptosis and ferroptosis. Am. J. Respir. Cell Mol. Biol. 62 (5), 554–562. 10.1165/rcmb.2019-0337TR 32017592

[B56] Muszyński P. Bonda T. A. (2021). Mitochondrial dysfunction in atrial fibrillation-mechanisms and pharmacological interventions. J. Clin. Med. 10 (11), 2385. 10.3390/jcm10112385 34071563 PMC8199309

[B57] Nattel S. (2013). Paroxysmal atrial fibrillation and pulmonary veins: relationships between clinical forms and automatic versus re-entrant mechanisms. Can. J. Cardiol. 29 (10), 1147–1149. 10.1016/j.cjca.2013.07.797 23993351

[B58] Nattel S. (2018). Electrical coupling between cardiomyocytes and fibroblasts: experimental testing of a challenging and important concept. Cardiovasc Res. 114 (3), 349–352. 10.1093/cvr/cvy003 29360945 PMC6018686

[B59] Nattel S. Heijman J. Zhou L. Dobrev D. (2020). Molecular basis of atrial fibrillation pathophysiology and therapy: a translational perspective. Circ. Res. 127 (1), 51–72. 10.1161/CIRCRESAHA.120.316363 32717172 PMC7398486

[B60] Nguyen T. P. Qu Z. Weiss J. N. (2014). Cardiac fibrosis and arrhythmogenesis: the road to repair is paved with perils. J. Mol. Cell Cardiol. 70, 83–91. 10.1016/j.yjmcc.2013.10.018 24184999 PMC3995831

[B61] Ongstad E. Kohl P. (2016). Fibroblast-myocyte coupling in the heart: potential relevance for therapeutic interventions. J. Mol. Cell Cardiol. 91, 238–246. 10.1016/j.yjmcc.2016.01.010 26774702 PMC5022561

[B62] Pandit S. V. Jalife J. (2013). Rotors and the dynamics of cardiac fibrillation. Circ. Res. 112 (5), 849–862. 10.1161/CIRCRESAHA.111.300158 23449547 PMC3650644

[B63] Park E. Chung S. W. (2019). ROS-mediated autophagy increases intracellular iron levels and ferroptosis by ferritin and transferrin receptor regulation. Cell Death Dis. 10 (11), 822. 10.1038/s41419-019-2064-5 31659150 PMC6817894

[B64] Peng H. Fu S. Wang S. Xu H. Dhanasekaran M. Chen H. (2022). Ablation of FUNDC1-dependent mitophagy renders myocardium resistant to paraquat-induced ferroptosis and contractile dysfunction. Biochim. Biophys. Acta Mol. Basis Dis. 1868 (9), 166448. 10.1016/j.bbadis.2022.166448 35598771

[B65] Piccini J. P. Hammill B. G. Sinner M. F. Jensen P. N. Hernandez A. F. Heckbert S. R. (2012). Incidence and prevalence of atrial fibrillation and associated mortality among Medicare beneficiaries, 1993-2007. Circ. Cardiovasc Qual. Outcomes 5 (1), 85–93. 10.1161/CIRCOUTCOMES.111.962688 22235070 PMC3332107

[B66] Pickles S. Vigié P. Youle R. J. (2018). Mitophagy and quality control mechanisms in mitochondrial maintenance. Curr. Biol. 28 (4), R170–R185. 10.1016/j.cub.2018.01.004 29462587 PMC7255410

[B67] Platonov P. G. Mitrofanova L. B. Orshanskaya V. Ho S. Y. (2011). Structural abnormalities in atrial walls are associated with presence and persistency of atrial fibrillation but not with age. J. Am. Coll. Cardiol. 58 (21), 2225–2232. 10.1016/j.jacc.2011.05.061 22078429

[B68] Protchenko O. Baratz E. Jadhav S. Li F. Shakoury-Elizeh M. Gavrilova O. (2021). Iron chaperone poly rC binding protein 1 protects mouse liver from lipid peroxidation and steatosis. Hepatology 73 (3), 1176–1193. 10.1002/hep.31328 32438524 PMC8364740

[B69] Riegman M. Sagie L. Galed C. Levin T. Steinberg N. Dixon S. J. (2020). Ferroptosis occurs through an osmotic mechanism and propagates independently of cell rupture. Nat. Cell Biol. 22 (9), 1042–1048. 10.1038/s41556-020-0565-1 32868903 PMC7644276

[B70] Rose R. A. Sellan M. Simpson J. A. Izaddoustdar F. Cifelli C. Panama B. K. (2011). Iron overload decreases CaV1.3-dependent L-type Ca2+ currents leading to bradycardia, altered electrical conduction, and atrial fibrillation. Circ. Arrhythm. Electrophysiol. 4 (5), 733–742. 10.1161/CIRCEP.110.960401 21747058 PMC3401539

[B71] Sahin E. Colla S. Liesa M. Moslehi J. Müller F. L. Guo M. (2011). Telomere dysfunction induces metabolic and mitochondrial compromise. Nature 470 (7334), 359–365. 10.1038/nature09787 21307849 PMC3741661

[B72] Schaper J. Meiser E. Stämmler G. (1985). Ultrastructural morphometric analysis of myocardium from dogs, rats, hamsters, mice, and from human hearts. Circ. Res. 56 (3), 377–391. 10.1161/01.res.56.3.377 3882260

[B73] Schnabel R. B. Sullivan L. M. Levy D. Pencina M. J. Massaro J. M. D'Agostino R. B. Sr (2009). Development of a risk score for atrial fibrillation (Framingham Heart Study): a community-based cohort study. Lancet 373 (9665), 739–745. 10.1016/S0140-6736(09)60443-8 19249635 PMC2764235

[B74] Seiler A. Schneider M. Förster H. Roth S. Wirth E. K. Culmsee C. (2008). Glutathione peroxidase 4 senses and translates oxidative stress into 12/15-lipoxygenase dependent- and AIF-mediated cell death. Cell Metab. 8 (3), 237–248. 10.1016/j.cmet.2008.07.005 18762024

[B75] Shen Z. Liu T. Li Y. Lau J. Yang Z. Fan W. (2018). Fenton-reaction-acceleratable magnetic nanoparticles for ferroptosis therapy of orthotopic brain tumors. ACS Nano 12 (11), 11355–11365. 10.1021/acsnano.8b06201 30375848

[B76] Stockwell B. R. Jiang X. Gu W. (2020). Emerging mechanisms and disease relevance of ferroptosis. Trends Cell Biol. 30 (6), 478–490. 10.1016/j.tcb.2020.02.009 32413317 PMC7230071

[B77] Strauss E. Tomczak J. Staniszewski R. Oszkinis G. (2018). Associations and interactions between variants in selenoprotein genes, selenoprotein levels and the development of abdominal aortic aneurysm, peripheral arterial disease, and heart failure. PLoS One 13 (9), e0203350. 10.1371/journal.pone.0203350 30188935 PMC6126836

[B78] Summermatter S. Santos G. Pérez-Schindler J. Handschin C. (2013). Skeletal muscle PGC-1α controls whole-body lactate homeostasis through estrogen-related receptor α-dependent activation of LDH B and repression of LDH A. Proc. Natl. Acad. Sci. U. S. A. 110 (21), 8738–8743. 10.1073/pnas.1212976110 23650363 PMC3666691

[B79] Sun D. Wang J. Toan S. Muid D. Li R. Chang X. (2022). Molecular mechanisms of coronary microvascular endothelial dysfunction in diabetes mellitus: focus on mitochondrial quality surveillance. Angiogenesis 25 (3), 307–329. 10.1007/s10456-022-09835-8 35303170

[B80] Tang D. Chen X. Kang R. Kroemer G. (2021). Ferroptosis: molecular mechanisms and health implications. Cell Res. 31 (2), 107–125. 10.1038/s41422-020-00441-1 33268902 PMC8026611

[B81] Tang D. Kang R. Berghe T. V. Vandenabeele P. Kroemer G. (2019). The molecular machinery of regulated cell death. Cell Res. 29 (5), 347–364. 10.1038/s41422-019-0164-5 30948788 PMC6796845

[B82] Thomas J. P. Geiger P. G. Maiorino M. Ursini F. Girotti A. W. (1990). Enzymatic reduction of phospholipid and cholesterol hydroperoxides in artificial bilayers and lipoproteins. Biochim. Biophys. Acta 1045 (3), 252–260. 10.1016/0005-2760(90)90128-k 2386798

[B83] Tomaszuk-Kazberuk A. Koziński M. Kuźma Ł. Bujno E. Łopatowska P. Rogalska E. (2020). Atrial fibrillation is more frequently associated with nonobstructive coronary lesions: the Bialystok Coronary Project. Pol. Arch. Intern Med. 130 (12), 1029–1036. 10.20452/pamw.15635 33016687

[B84] Van Wagoner D. R. Chung M. K. (2018). Inflammation, inflammasome activation, and atrial fibrillation. Circulation 138 (20), 2243–2246. 10.1161/CIRCULATIONAHA.118.036143 30571523 PMC6334772

[B85] Vlachos K. Letsas K. P. Korantzopoulos P. Liu T. Georgopoulos S. Bakalakos A. (2016). Prediction of atrial fibrillation development and progression: current perspectives. World J. Cardiol. 8 (3), 267–276. 10.4330/wjc.v8.i3.267 27022458 PMC4807315

[B86] Voigt N. Heijman J. Wang Q. Chiang D. Y. Li N. Karck M. (2014). Cellular and molecular mechanisms of atrial arrhythmogenesis in patients with paroxysmal atrial fibrillation. Circulation 129 (2), 145–156. 10.1161/CIRCULATIONAHA.113.006641 24249718 PMC4342412

[B87] Wang H. L. Zhou X. H. Li Z. Q. Fan P. Zhou Q. N. Li Y. D. (2017). Prevention of atrial fibrillation by using sarcoplasmic reticulum calcium ATPase pump overexpression in a rabbit model of rapid atrial pacing. Med. Sci. Monit. 23, 3952–3960. 10.12659/msm.904824 28811460 PMC5569926

[B88] Wang S. Li F. Qiao R. Hu X. Liao H. Chen L. (2018). Arginine-rich manganese silicate nanobubbles as a ferroptosis-inducing agent for tumor-targeted theranostics. ACS Nano 12 (12), 12380–12392. 10.1021/acsnano.8b06399 30495919

[B89] Wasmer K. Eckardt L. Breithardt G. (2017). Predisposing factors for atrial fibrillation in the elderly. J. Geriatr. Cardiol. 14 (3), 179–184. 10.11909/j.issn.1671-5411.2017.03.010 28592961 PMC5460064

[B90] Wiersma M. van Marion D. M. S. Wüst R. C. I. Houtkooper R. H. Zhang D. Groot N. M. S. d. (2019). Mitochondrial dysfunction underlies cardiomyocyte remodeling in experimental and clinical atrial fibrillation. Cells 8 (10), 1202. 10.3390/cells8101202 31590355 PMC6829298

[B91] Williams B. A. Chamberlain A. M. Blankenship J. C. Hylek E. M. Voyce S. (2020). Trends in atrial fibrillation incidence rates within an integrated health care delivery system, 2006 to 2018. JAMA Netw. Open 3 (8), e2014874. 10.1001/jamanetworkopen.2020.14874 32857147 PMC7455855

[B92] Xie Y. Hou W. Song X. Yu Y. Huang J. Sun X. (2016). Ferroptosis: process and function. Cell Death Differ. 23 (3), 369–379. 10.1038/cdd.2015.158 26794443 PMC5072448

[B93] Xu T. Ding W. Ji X. Ao X. Liu Y. Yu W. (2019). Molecular mechanisms of ferroptosis and its role in cancer therapy. J. Cell Mol. Med. 23 (8), 4900–4912. 10.1111/jcmm.14511 31232522 PMC6653007

[B94] Yaegashi T. Kato T. Usui S. Kanamori N. Furusho H. Takashima S. I. (2016). Short-term rapid atrial pacing alters the gene expression profile of rat liver: cardiohepatic interaction in atrial fibrillation. Heart rhythm. 13 (12), 2368–2376. 10.1016/j.hrthm.2016.08.036 27574983

[B95] Yamashita T. Arima Y. Hoshiyama T. Tabata N. Sueta D. Kawahara Y. (2022). Effect of the *ALDH2* variant on the prevalence of atrial fibrillation in habitual drinkers. JACC Asia 2 (1), 62–70. 10.1016/j.jacasi.2021.10.009 36340257 PMC9627901

[B96] Yorimitsu T. Klionsky D. J. (2005). Autophagy: molecular machinery for self-eating. Cell Death Differ. 12 (Suppl. 2), 1542–1552. 10.1038/sj.cdd.4401765 16247502 PMC1828868

[B97] Yu L. M. Dong X. Huang T. Zhao J. K. Zhou Z. J. Huang Y. T. (2023). Inhibition of ferroptosis by icariin treatment attenuates excessive ethanol consumption-induced atrial remodeling and susceptibility to atrial fibrillation, role of SIRT1. Apoptosis 28 (3-4), 607–626. 10.1007/s10495-023-01814-8 36708428

[B98] Yue L. Xie J. Nattel S. (2011). Molecular determinants of cardiac fibroblast electrical function and therapeutic implications for atrial fibrillation. Cardiovasc Res. 89 (4), 744–753. 10.1093/cvr/cvq329 20962103 PMC3039247

[B99] Yue Z. Xie J. Yu A. S. Stock J. Du J. Yue L. (2015). Role of TRP channels in the cardiovascular system. Am. J. Physiol. Heart Circ. Physiol. 308 (3), H157–H182. 10.1152/ajpheart.00457.2014 25416190 PMC4312948

[B100] Zheng J. Conrad M. (2020). The metabolic underpinnings of ferroptosis. Cell Metab. 32 (6), 920–937. 10.1016/j.cmet.2020.10.011 33217331

[B101] Zhu M. Peng L. Huo S. Peng D. Gou J. Shi W. (2023). STAT3 signaling promotes cardiac injury by upregulating NCOA4-mediated ferritinophagy and ferroptosis in high-fat-diet fed mice. Free Radic. Biol. Med. 201, 111–125. 10.1016/j.freeradbiomed.2023.03.003 36940731

[B102] Zhu Y. Gu Z. Shi J. Chen C. Xu H. Lu Q. (2022b). Vaspin attenuates atrial abnormalities by promoting ULK1/FUNDC1-mediated mitophagy. Oxid. Med. Cell Longev. 15, 3187463. 10.1155/2022/3187463 PMC9681551 36425056

[B103] Zhu Z. Y. Liu Y. D. Gong Y. Jin W. Topchiy E. Turdi S. (2022a). Mitochondrial aldehyde dehydrogenase (ALDH2) rescues cardiac contractile dysfunction in an APP/PS1 murine model of Alzheimer's disease via inhibition of ACSL4-dependent ferroptosis. Acta Pharmacol. Sin. 43 (1), 39–49. 10.1038/s41401-021-00635-2 33767380 PMC8724276

[B104] Zilka O. Shah R. Li B. Friedmann Angeli J. P. Griesser M. Conrad M. (2017). On the mechanism of cytoprotection by ferrostatin-1 and liproxstatin-1 and the role of lipid peroxidation in ferroptotic cell death. ACS Cent. Sci. 3 (3), 232–243. 10.1021/acscentsci.7b00028 28386601 PMC5364454

[B105] Zoni-Berisso M. Lercari F. Carazza T. Domenicucci S. (2014). Epidemiology of atrial fibrillation: European perspective. Clin. Epidemiol. 6, 213–220. 10.2147/CLEP.S47385 24966695 PMC4064952

